# Association between Geriatric Nutritional Risk Index and Mortality in Older Trauma Patients in the Intensive Care Unit

**DOI:** 10.3390/nu12123861

**Published:** 2020-12-17

**Authors:** Hang-Tsung Liu, Shao-Chun Wu, Ching-Hua Tsai, Chi Li, Sheng-En Chou, Wei-Ti Su, Shiun-Yuan Hsu, Ching-Hua Hsieh

**Affiliations:** 1 Department of Trauma Surgery, Kaohsiung Chang Gung Memorial Hospital, Chang Gung University College of Medicine, Kaohsiung City 833, Taiwan; htl1688@yahoo.com.tw (H.-T.L.); tsai1737@cloud.cgmh.org.tw (C.-H.T.); foocollie7@gmail.com (C.L.); athenechou@gmail.com (S.-E.C.); s101132@adm.cgmh.org.tw (W.-T.S.); ah.lucy@hotmail.com (S.-Y.H.); 2 Department of Anesthesiology, Kaohsiung Chang Gung Memorial Hospital, Chang Gung University College of Medicine, Kaohsiung City 833, Taiwan; shaochunwu@gmail.com; 3 Department of Plastic Surgery, Kaohsiung Chang Gung Memorial Hospital, Chang Gung University College of Medicine, Kaohsiung City 833, Taiwan

**Keywords:** trauma, intensive care unit, mortality, malnutrition, elderly, the geriatric nutritional risk index

## Abstract

The geriatric nutritional risk index (GNRI) is a simple and efficient tool to assess the nutritional status of patients with malignancies or after surgery. Because trauma patients constitute a specific population that generally acquires accidental and acute injury, this study aimed to identify the association between the GNRI at admission and mortality outcomes of older trauma patients in the intensive care unit (ICU). Methods: The study population included 700 older trauma patients admitted to the ICU between 1 January 2009 and 31 December 2019. The collected data included age, sex, body mass index (BMI), albumin level at admission, preexisting comorbidities, injury severity score (ISS), and in-hospital mortality. Multivariate logistic regression analysis was conducted to identify the independent effects of univariate predictive variables resulting in mortality in our study population. The study population was categorized into four nutritional risk groups: a major-risk group (GNRI < 82; *n* = 128), moderate-risk group (GNRI 82 to <92; *n* = 191), low-risk group (GNRI 92–98; *n* = 136), and no-risk group (GNRI > 98; *n* = 245). Results: There was no significant difference in sex predominance, age, and BMI between the mortality (*n* = 125) and survival (*n* = 575) groups. The GNRI was significantly lower in the mortality group than in the survival group (89.8 ± 12.9 vs. 94.2 ± 12.0, *p* < 0.001). Multivariate logistic regression analysis showed that the GNRI (odds ratio—OR, 0.97; 95% confidence interval (CI) 0.95–0.99; *p* = 0.001), preexisting end-stage renal disease (OR, 3.6; 95% CI, 1.70–7.67; *p* = 0.001), and ISS (OR, 1.1; 95% CI, 1.05–1.10; *p* < 0.001) were significant independent risk factors for mortality. Compared to the patients in group of GNRI > 98, those patients in group of GNRI < 82 presented a significantly higher mortality rate (26.6% vs. 13.1%; *p* < 0.001) and length of stay in hospital (26.5 days vs. 20.9 days; *p* = 0.016). Conclusions: This study demonstrated that GNRI is a significant independent risk factor and a promising simple screening tool to identify the subjects with malnutrition associated with higher risk for mortality in those ICU elderly trauma patients.

## 1. Background

The prevalence of malnutrition in hospitalized patients ranges from 10% to 50%, depending on the study population and diagnosis criteria [1]. In patients aged >65 years, malnutrition appears to be a common problem [2], especially in older hospitalized patients [3]. However, malnutrition is generally unrecognized and not treated properly in hospitalized patients [4]. For the critically ill patients in the intensive care unit (ICU), their nutritional status deteriorates rapidly after admission due to stress-related severe catabolism and the effects of malnutrition are likely to be more magnified [5,6]. In a systemic review of 20 studies, malnutrition diagnosed by nutrition assessments was independently associated with increased in ICU length of stay (LOS), ICU readmission, incidence of infection, and in-hospital mortality rate [7]. Using appropriate nutrition screening and assessment tools will help identify effective strategies that reduce the negative impact of malnutrition [8,9]. Therefore, for those elderly patients admitted into the ICU, it is important to identify patients at risk of malnutrition early and to treat them adequately.

There are several methods for assessing the nutritional status, such as albumin level, body mass index (BMI), muscle circumference, prognostic nutritional index (based on the serum albumin level and peripheral blood lymphocyte count), and questionnaires. The serum albumin level can be modified by inflammatory processes, hydration, and hepatic or renal impairment [10]; therefore, serum albumin is considered a better marker of inflammation and severity of acute illness than nutritional status [11]. Furthermore, anthropometric parameters such as BMI, weight loss, muscle circumferences, and skinfold thicknesses do not reflect the actual nutritional status of patients when applied separately [12]. The nutritional risk index (NRI) is a screening method that was primarily developed to identify older patients with malnutrition [13]. It consists of serum albumin levels as well as body weight measurements. However, even under professional care, the usual body weight is often not documented for older patients [14]. To determine the usual body weight of older patients, the geriatric nutritional risk index (GNRI) was introduced in 2005 by Bouillanne et al. [15]—the formula included a combination of serum albumin levels and the ratio of body weight to ideal body weight. The GNRI formula replaces the usual body weight in the NRI formula with the ideal body weight, calculated using the Lorentz formula [15]. The ratio of body weight to ideal body weight used in the GNRI might reflect the degree of frailty and cachexia associated with a poor prognosis in older patients [16]. Thus, the GNRI, which combines the factors of albumin and body weight status, may predict nutrition-related risk better than the serum albumin level or BMI [15]. The GNRI has been found to be superior to the albumin level and BMI, when used separately, in predicting cardiovascular-related mortality [17]. On the other hand, The Subjective Global Assessment classifies patients using information on the history of illness and physical examination [18]. However, the tool is too complex and is not suitable for rapid screening purposes [19]. The Mini Nutritional Assessment (MNA) relies on the completeness of its questionnaire and thus cannot be used with older patients who have difficulty communicating, for example, intubated patients in the ICU. Because it requires only objective parameters that can be readily collected and does not depend on a caregiver or memory, the GNRI is clearly less time consuming than other questionnaire-based assessment procedures and requires minimal participation by patients [2,15], thus being suitable for older patients with critical illness in the ICU. The GNRI may also be useful for older patients with cognitive impairment or delirium [20]. In addition, the GNRI score was found to be superior to the Mini Nutritional Assessment-Short Form (MNA–SF) score for risk discrimination regarding the overall survival in cancer patients [21].Although the GNRI was developed using the data of patients who were admitted to a geriatric rehabilitation care unit [15], it was found to be a strong prognostic factor for certain malignancies [22,23] and a simple, objective, and quick method to determine the nutritional status of patients and long-term postoperative outcomes and the correlation between these elements [24,25,26,27,28]. Using this simple calculation, it is possible to evaluate the nutritional status of critically ill patients with acute respiratory distress syndrome in the ICU [29].

Considering that trauma patients constitute a specific population, as injuries generally occur accidentally and acutely, it is important to determine whether the GNRI can be used to link nutritional status and outcomes in trauma patients. Therefore, this study aimed to identify the association between GNRI at admission and mortality outcomes of older trauma patients in the ICU.

## 2. Methods

### 2.1. Ethics Statement

This study was approved by the Institutional Review Board (IRB) of Chang Gung Memorial Hospital (approval number 202001446B0). Because the study was designed for retrospective analysis of the registered database, the need for informed consent was waived according to IRB regulations.

### 2.2. Study Population and Data Collection

**Figure 1:**
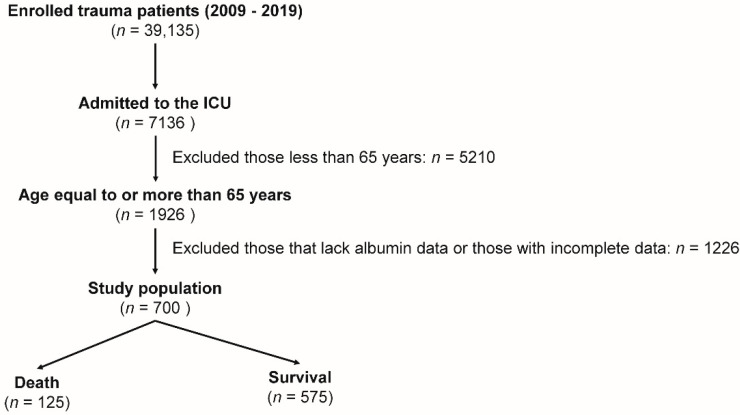
Flow chart illustrating the inclusion of older trauma patients in the ICU and the allocation of these patients into the mortality group and survival group.

The medical records of 39,135 enrolled trauma patients registered between 1 January 2009 and 31 December 2019 were reviewed for this study (Figure 1). The enrolled patients experienced trauma from different injuries and were hospitalized for treatment. Of the 7136 patients who were admitted to the ICU, 1926 older trauma patients were aged ≥65 years. After excluding patients whose albumin data were not available and those with incomplete data (*n* = 1226), 700 older trauma patients were finally included in the study. The study population was categorized according to the original description provided by Bouillanne et al. [15] into four nutritional risk groups: a major-risk group (GNRI < 82; *n* = 128), moderate-risk group (GNRI 82 to <92; *n* = 191), low-risk group (GNRI 92–98; *n* = 136), and no-risk group (GNRI > 98; *n* = 245). Detailed information of the study population was extracted from the Trauma Registry System of the hospital [30,31,32]. The following data were collected: age, sex, body mass index (BMI), albumin level at admission, preexisting comorbidities (diabetes mellitus—DM, hypertension—HTN, coronary artery disease—CAD, congestive heart failure—CHF, cerebral vascular accident—CVA, end-stage renal disease—ESRD, and chronic obstructive pulmonary disease—COPD), injury severity score (ISS), and in-hospital mortality. The 1998 version of the Abbreviated Injury Scale (AIS) was used to record scores [33]. The AIS measures injury severity of a trauma patient with a numeric method for ranking anatomy-based specific injuries [1], with the severity of the anatomical injury assessed on a six-point ordinal scale ranging from minor (1), moderate (2), serious (3), severe (4), critical (5), to un-survivable injury (6) [34,35]. ISS, which represents the severity of injury in patients with multiple injuries and ranges from 1 to 75, was calculated using the sum of the squares of the three highest AIS scores in different body regions [36,37]. The GNRI was calculated using the albumin level and the ratio of body weight to ideal body weight as per the following formula: −1.489 × albumin (g/dL) + 41.7 × (body weight/ideal body weight). The ideal body weight of men is (body height in cm − 80) × 0.7, and that of women is (body height in cm − 70) × 0.6.

### 2.3. Statistical Analyses

In this study, all statistical analyses were performed using Window version 23.0 for SPSS (IBM Inc., Chicago, IL, USA). Two-sided Fisher’s exact test or Pearson χ^2^ test was used to compare categorical data. The normalization of the distributed data for continuous variables was assessed using the Kolmogorov–Smirnov test. Unpaired Student’s *t*-test and the Mann–Whitney *U* test were used to analyze normally and non-normally distributed continuous data, respectively. The results are expressed as mean ± standard deviation, with ISS presented as median and interquartile range (IQR, Q1–Q3). Multivariate logistic regression analysis was conducted to identify the independent effects of univariate predictive variables leading to mortality in older patients with trauma. The odds ratios (ORs) of the risk factors associated with mortality and their 95% confidence intervals (CIs) were calculated with post hoc correction. *p* values < 0.05 were considered significant.

## 3. Results

### 3.1. Patient Demographics

As shown in Table 1, the study population was categorized into two groups: mortality (*n* = 125) and survival (*n* = 575). There was no significant difference in sex predominance, age, and BMI between the two groups. The albumin level and GNRI were significantly lower in the mortality group than in the survival group (albumin level: 3.0 ± 0.8 vs. 3.3 ± 0.6, *p* < 0.001; GNRI: 89.8 ± 12.9 vs. 94.2 ± 12.0, *p* < 0.001). There were no significant intergroup differences in the prevalence of preexisting comorbidities, except for a significantly lower rate of HTN (47.2% vs. 57.9%, *p* = 0.029) and higher rate of ESRD (10.4% vs. 4.2%, *p* = 0.005) in the mortality group than in the survival group. A significantly higher ISS was found in the group with fatal injuries than in the survival group (median—IQR: 25 [16,17,18,19,20,21,22,23,24,25,26,27,28,29] vs. 16 [13,14,15,16,17,18,19,20,21,22,23,24,25], *p* < 0.001). When stratified by ISS (1–15, 16–24, or ≥ 25), significantly fewer fatal patients had an ISS of 1–15 and 16–24 and more fatal patients had an ISS of ≥25 than survival patients. The patients in the mortality group had a shorter hospital LOS (19.7 days vs. 24.8 days, *p* = 0.006) than those in the survival group.

### 3.2. Risk Factors for Mortality

Univariate logistic regression analysis (Table 2) showed that the significant risk factors for mortality in older trauma patients in the ICU were the GNRI, preexisting HTN and ESRD, and ISS. Multivariate logistic regression analysis revealed that the GNRI (OR, 0.97; 95% CI, 0.95–0.99; *p* = 0.001), preexisting ESRD (OR, 3.6; 95% CI, 1.70–7.67; *p* = 0.001), and ISS (OR, 1.1; 95% CI, 1.05–1.10; *p* < 0.001) were significant independent risk factors for mortality. Gender (OR, 0.9; 95% CI, 0.57–1.33; *p* = 0.511), age (OR, 1.0; 95% CI, 0.99–1.05; *p* = 0.129), and preexisting HTN (OR, 0.7; 95% CI, 0.47–1.09; *p* = 0.120) was not recognized as a significant independent risk factor for mortality in older trauma patients in the ICU.

### 3.3. Comparison of Patients with Low and High GNRI

As shown in Table 3, the patients in group of GNRI <82 were significantly older than the patients in group of GNRI >98. The patients in groups of GNRI <82, 82 to <92, and 92 to ≤98 presented a significantly lower BMI and level of albumin than the patients in group of GNRI >98. There were no significant intergroup differences in the prevalence of preexisting comorbidities, except HTN. There was no significant difference in the ISS among these groups of patients regardless of ISS stratification (1–15, 16–24, and ≥25). Compared to the patients in group of GNRI >98, those patients in group of GNRI < 82 presented a significantly higher mortality rate (26.6% vs. 13.1%; *p* < 0.001) and LOS in hospital (26.5 days vs. 20.9 days; *p* = 0.012). In contrast, no significant differences of mortality rate and LOS in hospital were found in those patients in groups of GNRI of 82 to <92 and of 92 to ≤98 than those patients in groups of GNRI >98.

## 4. Discussion

In this study, multivariable logistic regression analysis identified the GNRI as an independent predictor of mortality in older trauma patients in the ICU. Although the odds ratio for mortality is small with the GNRI (OR 0.97; 95% CI, 0.95–0.99), the estimate is in accordance with the results showing that the GNRI was significantly lower in the mortality group than in the survival group and that the mortality rate was significantly higher in patients with low GNRI than in those with high GNRI. The GNRI has been validated by its correlation to indexes obtained from other nutritional scoring systems [10,11]. A strong association between the GNRI, mid-upper arm muscle circumference, arm muscle area, and handgrip strength in hospitalized patients [38] as well as with preoperative sarcopenia status in patients with cancer has been reported [39]. Malnourished patients are at a higher risk of developing postoperative complications, which in turn may affect their prognosis, leading to decreased survival rates [40,41].

In the original study by Bouillanne et al. [15], the GNRI scores were categorized into four nutrition-related risk groups (high-risk: GNRI < 82, moderate-risk: GNRI = 82 to <92, low-risk: GNRI = 92–98, and very low-risk: GNRI > 98), and the risk of infectious complications or mortality was significantly higher in the high-, moderate-, and low-risk groups than in the very low-risk group [7]. For older patients with sepsis, the odds of mortality for each GNRI group were reported to be 11.6-, 5.8-, and 2.3-fold times higher in the high-, moderate-, and low-risk groups, respectively, than in the very low-risk group [42]. In this study, the results demonstrated that the GNRI helps identify major risk-malnutrition elderly trauma patients in the ICU. Compared to the patients in group of GNRI > 98, those patients in group of GNRI < 82 presented a significantly higher mortality rate and LOS in hospital. Therefore, in the ICU, the group of elderly trauma patients with GNRI < 82 would require specific attention regarding their nutritional status. Some authors have proposed that a GNRI value of less than 87 is significantly associated with mortality in critically ill cancer patients [43]. While the GNRI measure is relevant for prognosis, the optimal GNRI cutoff values remain to be elucidated for older trauma patients with critical illnesses. Furthermore, preoperative nutritional interventions help patients cope with surgical stress and reduce the risk of postoperative complications [44]. Furthermore, high-risk GNRI patients can benefit from methods that aim to ameliorate the nutritional and functional status of cancer patients [45]. However, the effect of nutritional intervention in patients with high-risk GNRI remains to be validated.

Some of the limitations of this study are as follows: First, data of patients declared dead on arrival at the emergency room were not recorded in the registered database and only in-hospital mortality was evaluated. Second, selection bias may have been induced by the retrospective design of this study. Unknown conditions such as resuscitation, damage control, and surgical intervention could lead to bias. We assumed that the outcome of treatments was uniform across the studied population. Third, this study excluded many patients who had no albumin data or had incomplete data, and such a scenario may lead to selection bias. Fourth, the population included in this study was limited to that from a single urban trauma center in southern Taiwan; thus, these results may not be generalizable to other regions.

## 5. Conclusions

This study demonstrated that GNRI is a significant independent risk factor and a promising simple screening tool to identify the subjects with malnutrition associated with higher risk for mortality in those ICU elderly trauma patients.

## Figures and Tables

**Table 1:** Patient and injury characteristics of the mortality and survival groups of older trauma patients in the intensive care unit.

Variables	Death *n* = 125	Survival *n* = 575	*p*-Value
Gender			0.696
Male, *n* (%)	79 (63.2)	374 (65.0)	
Female, *n* (%)	46 (36.8)	201 (35.0)	
Age (years)	76.4 ± 7.5	75.7 ± 7.0	0.386
BMI	23.6 ± 4.5	23.4 ± 4.0	0.604
Albumin (g/dl)	3.0 ± 0.8	3.3 ± 0.6	<0.001
GNRI	89.8 ± 12.9	94.2 ± 12.0	<0.001
Co-morbidities			
DM, *n* (%)	39 (31.2)	165 (28.7)	0.577
HTN, *n* (%)	59 (47.2)	333 (57.9)	0.029
CAD, *n* (%)	22 (17.6)	83 (14.4)	0.369
CHF, *n* (%)	4 (3.2)	12 (2.1)	0.450
CVA, *n* (%)	12 (9.6)	58 (10.1)	0.869
ESRD, *n* (%)	13 (10.4)	24 (4.2)	0.005
COPD, *n* (%)	4 (3.2)	19 (3.3)	0.953
ISS, median (IQR)	25 (16–29)	16 (13–25)	<0.001
1–15, *n* (%)	12 (9.6)	158 (27.5)	<0.001
16–24, *n* (%)	44 (35.2)	267 (46.4)	0.022
≥25, *n* (%)	69 (55.2)	150 (26.1)	<0.001
LOS in hospital (days)	19.7 ± 20.9	24.8 ± 18.4	0.006

BMI = Body mass index; CAD = coronary artery disease; CHF = congestive heart failure; COPD = chronic obstructive pulmonary disease; CVA = cerebral vascular accident; DM = diabetes mellitus; ESRD = end-stage renal disease; GNRI =Geriatric Nutritional Risk Index; HTN = Hypertension; ISS = injury severity score; LOS = length of stay.

**Table 2:** Univariate and multivariate analysis to identify risk factors for mortality in older trauma patients in the intensive care unit.

Variables	Univariate Analysis		Multivariate Analysis	
	OR (95% CI)	*p*-Value	OR (95% CI)	*p*-Value
Gender	0.9 (0.62–1.38)	0.696	0.9 (0.57–1.33)	0.511
Age	1.0 (0.99–1.04)	0.385	1.0 (0.99–1.05)	0.129
GNRI	0.97 (0.95–0.99)	<0.001	0.97 (0.95–0.99)	0.001
HTN	0.7 (0.44–0.96)	0.029	0.7 (0.47–1.09)	0.120
ESRD	2.7 (1.32–5.39)	0.006	3.6 (1.70–7.67)	0.001
ISS	1.1 (1.05–1.10)	<0.001	1.1 (1.05–1.10)	<0.001

CI = confidence interval; ESRD = end-stage renal disease; GNRI = Geriatric Nutritional Risk Index; HTN = hypertension; ISS = injury severity score; OR = odds ratio.

**Table 3:** Patient characteristics and outcomes of the ICU elderly trauma patients with different risks of malnutrition according to GNRI.

GNRI: Variables	<82 *n* = 128	82 to <92 *n* = 191	92 to ≤98 *n* = 136	>98 *n* = 245	*p*-Value
Gender					0.862
Male, *n* (%)	86(67.2)	124(64.9)	89(65.4)	154(62.9)	
Female, *n* (%)	42(32.8)	67(35.1)	47(34.6)	91(37.1)	
Age (years)	78.2 ± 7.5 *	75.9 ± 7.5	76.1 ± 6.3	74.4 ± 6.7	<0.001
BMI	19.9 ± 2.7 *	21.7 ± 2.9 *	23.6 ± 2.9 *	26.5 ± 3.7	<0.001
Albumin (g/dl)	2.5 ± 0.6 *	3.0 ± 0.5 *	3.3 ± 0.5 *	3.8 ± 0.5	<0.001
Co-morbidities					
DM, *n* (%)	31(24.2)	51(26.7)	39(28.7)	83(33.9)	0.193
HTN, *n* (%)	57(44.5) *	99(51.8) *	79(58.1)	157(64.1)	0.002
CAD, *n* (%)	14(10.9)	30(15.7)	25(18.4)	36(14.7)	0.396
CHF, *n* (%)	3(2.3)	5(2.6)	5(3.7)	3(1.2)	0.474
CVA, *n* (%)	9(7.0)	19(9.9)	18(13.2)	24(9.8)	0.416
ESRD, *n* (%)	4(31.1)	12(6.3)	8(5.9)	13(5.3)	0.644
COPD, *n* (%)	6(4.7)	6(3.1)	5(3.7)	6(2.4)	0.703
ISS, median (IQR)	17(16–25)	17(16–25)	17(16–25)	16(13–25)	0.345
1–15, *n* (%)	31(24.2)	39(20.4)	28(20.6)	72(29.4)	0.110
16–24, *n* (%)	53(41.4)	92(48.2)	64(47.1)	102(41.6)	0.438
≥ 25, *n* (%)	44(34.4)	60(31.4)	44(32.4)	71(29.0)	0.742
Mortality, *n* (%)	34(26.6) *	37(19.4)	22(16.2)	32(13.1)	0.012
LOS in hospital (days)	26.5 ± 21.6 *	25.6 ± 18.7	24.4 ± 20.2	20.9 ± 16.7	0.020

BMI = Body mass index; CAD = coronary artery disease; CHF = congestive heart failure; CI = confidence interval; COPD = chronic obstructive pulmonary disease; CVA = cerebral vascular accident; DM = diabetes mellitus; ESRD = end-stage renal disease; GCS = Glasgow Coma Scale; HTN = hypertension; IQR = interquartile range; ISS = injury severity score; LOS = length of stay; OR = odds ratio. * indicate *p* < 0.05 when compared with those patients with GNRI > 98.
